# Human iPS Cell-Derived Cardiac Tissue Sheets: a Platform for Cardiac Regeneration

**DOI:** 10.1007/s11936-016-0489-z

**Published:** 2016-09-16

**Authors:** Hidetoshi Masumoto, Jun K. Yamashita

**Affiliations:** 1 Department of Stem Cell Growth and Differentiation, Center for iPS Cell Research and Application (CiRA), Kyoto University, 53 Shogoin Kawahara-cho, Sakyo-ku, Kyoto 606-8507 Japan; 2 Department of Cardiovascular Surgery, Kyoto University Graduate School of Medicine, Kyoto, Japan

**Keywords:** Cardiac tissue sheets, Myocardial infarction, Stem cell therapy, Heart disease

## Abstract

Stem cell therapy is a promising therapeutic option for severe cardiac diseases that are resistant to conventional therapies. To overcome the unsatisfactory results of most clinical researches on stem cell injections to an injured heart, we are developing bioengineered cardiac tissue grafts using pluripotent stem cell-derived cardiomyocytes and vascular cells. We have validated the functional benefits of mouse embryonic stem cell-derived and human induced pluripotent stem cell-derived cardiac tissue sheets (CTSs) in a rat myocardial infarction model. We further showed enhanced functional recovery and engraftment efficiency leading to de novo myocardium upon transplanting thick multi-layered CTSs that had gelatin hydrogel microspheres between the layers. We anticipate that the combination of pluripotent stem cell biology and tissue engineering will contribute to future stem cell therapies for severe heart diseases.

## Introduction

Cardiovascular disease remains a major cause of death with increasing medical costs worldwide despite great advances in therapeutic modalities and risk-reduction strategies [1]. Myocardial infarction (MI) is a major cause of the mortality due to a massive loss of cardiomyocytes (CMs) and other cardiac cell types, which lead to scar formation and ventricular remodeling [2]. Percutaneous angioplasty and coronary artery bypass surgery are common approaches for recovering blood perfusion to the ischemic myocardium. However, these therapies do not promote myocardial regeneration in the injured heart and are less effective in patients with severe ischemic cardiomyopathy. This problem has prompted researchers to investigate new therapeutic strategies such as regenerative therapy for severe cardiac diseases that are resistant to conventional therapeutic approaches [3, 4].

Stem cell-based therapy is one promising strategy for myocardial restoration that ameliorates cardiac dysfunction through the secretion of paracrine factors and by replenishing the lost myocardium as a de novo myocardium [5]. The discovery of various stem cell populations possessing cardiogenic potential and subsequent technologies to isolate and expand these stem cell populations has already led to a number of clinical trials (Table 1) [26]. However, the direct injection of stem cells or their derivatives into ischemic hearts has failed to sufficiently improve cardiac function because the microenvironment of the ischemic heart does not adequately support survival of the grafted cells. It is reported that more than 70 % of cells will die during the first 48 h after direct injection, and even the surviving cells are progressively lost within the next several days due to the hypoxic, inflammatory, and/or fibrotic microenvironment [27]. Another report found that only 5.4–8.8 % of microspheres directly injected into a beating heart remain just after the injection due to massive physical loss [28]. The poor survival rates of the injected cells are one major reason why numerous clinical studies on cardiac stem cell therapies involving direct or catheter-based injections demonstrate only modest improvement [29]. The low survival percentage of the grafted cells diminishes the potential of this approach as an effective therapy.

**Table 1:** Stem cell populations used for clinical trials on heart diseases

Stem cell type	Origin	Clinical trial/transplantation method
Pluripotent stem cells		
Embryonic stem cells (ESCs)	Inner cell mass of the preimplantation blastocyst	ESCORT [6]/ESC-derived Isl-1^+^ SSEA-1^+^ cardiac progenitors embedded into a fibrin scaffold
Induced pluripotent stem cells (iPSCs)	Most somatic cells (e.g., skin fibroblasts, blood cells)	None
Bone-marrow derived stem cells		
Hematopoietic stem cells (circulating progenitor cells, bone marrow mononuclear cells)	Bone marrow, peripheral blood	BOOST [7, 8]/intracoronary injection REPAIR-AMI [9]/intracoronary injection TOPCARE-AMI [10, 11]/intracoronary injection FOCUS-CCTRN [12]/transendocardial injection TIME [13]/intracoronary injection LateTIME [14]/intracoronary injection BAMI [15]/intracoronary injection
Mesenchymal stem cells	Bone marrow (adherent cells), adipose tissue	TAC-HFT [16]/transendocardial injection POSEIDON [17]/transendocardial injection C-CURE [18]/transendocardial injection
Skeletal myoblasts	Mature skeletal muscle (between the sarcolemma and basement membrane)	MAGIC [19]/transepicardial injection CAuSMIC [20]/transepicardial injection A report from Japan [21]/cell sheet
Cardiac stem cells	Niches in the myocardium	SCIPIO [22, 23]/intracoronary injection; under concern about the integrity of data CADUCEUS [24]/intracoronary injection ALCADIA [25]/transepicardial injection

Overall, results from basic and clinical research studies concluded that stem cells may be beneficial as heart therapy, but act primarily through paracrine mechanisms, including angiogenesis, cell survival, anti-fibrosis and/or cell homing rather than through a direct contribution to the ventricular contractions of a regenerated myocardium [30]. To obtain better therapeutic outcome, new strategies that generate stem cell-derived, 3-dimensional (3D) cardiac tissues are desired. In this review, we introduce our strategy, which combines cardiovascular cell differentiation from human induced pluripotent stem (iPS) cells and bioengineered 3D cardiac tissues based on cell sheets, to effectively engraft transplants for stem cell-based cardiac regenerative therapy.

## Advantages of pluripotent stem cells in myocardial regeneration

Embryonic stem cells (ESCs) are pluripotent stem cells (PSCs) collected from the inner cell mass of the blastocyst and expanded in vitro [31]. Induced pluripotent stem cells (iPSCs) are another PSC population and were first reported by Yamanaka and colleagues, who reprogrammed adult somatic cells by activating four transcription factor genes to generate ESC-like cells [32, 33]. PSCs possess great capacity for cardiac regeneration due to several reasons explained below.

The first reason is that PSCs can be indefinitely expanded in vitro while retaining their pluripotency. In this regard, the regenerative capacity of PSCs is theoretically limitless [34]. The advantage of PSCs is especially great in therapy for heart diseases compared to other organs, such as endocrine or sensory organs, because the heart requires a large assembly of different cell types, including CMs and non-myocytes (e.g., vascular cells and cardiac fibroblasts). In fact, over a billion heart cells are estimated for cell therapies that fully compensate the damaged human heart tissue [35].

The second reason is their high capacity of differentiation toward any required cell types, such as CMs or other cardiovascular cell types. The differentiation of PSCs can be regulated by culture conditions such as monolayers or embryoid bodies in various growth media with or without serum [36, 37, 38•, 39]. We previously developed a novel monolayer culture-based cardiovascular cell differentiation system from mouse PSCs that recapitulates early cardiovascular developmental processes by generating Flk1 (also known as vascular endothelial cell growth factor [VEGF] receptor-2)-positive cells as a common cardiovascular progenitor. Cardiovascular cell types such as CMs [37], endothelial cells (ECs), and vascular mural cells (MCs, which describe cells that surround EC layers to support the vascular structure) [36] can be systematically induced and purified with this system. For human iPS cell differentiation toward CMs, several reports have shown the effectiveness of Wnt antagonists and inhibitors [40, 41]. In fact, of the various stem cell populations investigated so far, PSCs have demonstrated the greatest capacity for cardiac cell differentiation [42].

The third reason is the excellent potential of PSCs as models to elucidate cardiac regenerative mechanisms. As mentioned above, it has been recognized that transplanted adult stem cells work primarily through indirect paracrine mechanisms such as angiogenesis, cell survival, and so on. Consequently, the transplantation of somatic stem cells in basic and clinical studies has brought into question which stem cell populations are best. This is a particularly relevant question since derivatives from somatic stem cells like cardiosphere-derived cells might include heterogeneous cell populations in terms of cell lineage and differentiation stage. In this context, defined cardiovascular cell populations systematically induced from PSCs should be much more valuable than somatic stem cell-derived populations for the elucidation of cardiac regenerative mechanisms. The combination of different cell types in the transplant should help us elucidate the regenerative function of each cell type (for example, comparing the transplantation outcome of cell populations with and without CMs is helpful for elucidating the contribution of transplanted CMs to cardiac function) [43•].

The final reason is the discovery of iPSCs. iPSCs may resolve the ethical and immunogenic issues associated with ESCs. Regarding cardiac therapy, we have reported that cardiovascular cell types can be systematically differentiated from mouse iPSCs in almost the same manner as from mouse ESCs [44], suggesting iPSCs and ESCs hold similar cardiac regenerative capacities. Additionally, the establishment of an iPS cell bank that provides human leucocyte antigen-type matched and immunologically safe allogeneic iPSCs is expected to provide an off-the-shelf product that can further expand the clinical utility of iPSCs [45].

## Cell sheet technology using a temperature-responsive culture surface

By combining cells with injectable biomaterials such as fibrin, collagen or gelatin, investigators have demonstrated the use of biomaterials as scaffolds for cell transplantation. In addition, the use of a mixed extracellular matrix material (e.g., Matrigel® or similar material) provides a favorable environment for cell retention and survival that is abundant in cytokines and growth factors. In general, these early studies indicated improved survival of the transplanted cells and improved cardiac function [46]. However, these approaches did not assure sufficient cell retention or an adequate distribution of the transplanted cells. The generation of cell sheets or patches as micro-tissues without a scaffold support is now being investigated in order to achieve a more homogeneous and organized distribution of the transplanted cells and efficient survival [47]. Using this approach, inflammatory reactions against the biomaterials included in the scaffold might be avoided. As an example, Stevens et al. reported the transplantation of a scaffold-free, vascularized human cardiac tissue patch that consists of human ESC-derived CMs and ECs generated from a rotating orbital shaker-based culture method [48].

The generation of cell sheets based on two-dimensional cell culture is a more promising approach because of the larger scalability and accessibility. This method can be conducted with relative ease by utilizing a culture surface that is covalently grafted with temperature-responsive polymer poly (N-isopropylacrylamide) (PIPAAm), which allows us to collect cell sheets by simply decreasing the temperature and without enzymatic digestion [49]. The benefits of this method have been reported in many stem cell research studies, such as the transplantation of monolayer adipose tissue-derived mesenchymal stem cell sheets to an infarcted rat heart model [50], the transplantation of cell sheets made by mouse iPSC-derived genetically purified CMs to an infarcted mouse heart model [51] and a phase II clinical study of the transplantation of autologous skeletal myoblast sheets to patients with advanced heart failure due to ischemic etiology [21].

## Mouse ESC-derived cardiac tissue sheets

As mentioned above, we have established a novel cardiovascular cell differentiation system from mouse ES cells in which we can respectively induce and collect CMs and vascular cells in vitro [36, 37]. Utilizing the aforementioned temperature-responsive culture surface, we generated a self-pulsating cell sheet that consisted of mouse ESC-derived CMs, ECs and MCs, which we named “cardiac tissue sheet (CTS)” [43•]. We have transplanted three-layered CTSs onto an infarcted rat heart model and demonstrated the amelioration of cardiac dysfunction at 1 week after MI induction and confirmed that the mechanism of this functional advantage was mainly due to indirect paracrine effects. The accelerated neovascularization, mainly mediated by VEGF, was a key contributor to the attenuated ventricular remodeling. Furthermore, we introduced a cell sheet-based method to prospectively elucidate the cellular mechanisms of the cardiac functional restoration. Combinations of various cell types were transplanted as cell sheets, and we confirmed the incorporation of CMs within the transplanted cell population is indispensable for the functional improvement, mainly through neovascularization. We also found that vascular cells contribute to enhanced CTS function. Importantly, we can control the cellular composition of the PSC-derived sheet structures. These results support the future use of our mouse ESC-derived CTS system for elucidating cardiac regenerative mechanisms as well as for therapeutic purposes. Nevertheless, the efficiency of the engraftment of mouse ESC-derived CTSs was very low 4 weeks after transplantation, which encouraged us to investigate another cell source for better cell survival and myocardial regeneration.

## Human iPSC-derived cardiac tissue sheets

To realize the goal of PSC-mediated myocardial regeneration by supplying exogenous tissue to an injured heart, a potent PSC differentiation system toward CMs that has large scalability is required. Laflamme et al. reported a novel method to efficiently induce CMs from human ESCs using a serum-free, high-density monolayer culture that involved sequential treatment with TGFβ superfamily molecules such as Activin A (surrogating Nodal) and BMP4, which are important factors in embryonic heart development (directed differentiation) [52]. They successfully induced CMs from human ESCs with >50 times higher efficiency compared to conventional methods using embryonic bodies and medium including serum. We modified this protocol for human iPSC differentiation toward CMs using Dickkopf-1 (Dkk1), an antagonist of canonical Wnt signaling, which allowed us to establish a potent CM differentiation system from human iPSCs that consistently leads to CMs constituting 30–70 % of the total cell population [53].

As indicated in a cell-type controlled analysis using mouse ESCs [43•], supplying vascular cells along with CMs to a damaged heart might be crucial for cardiac functional restoration. We attempted to simultaneously induce ECs and MCs along with CMs by further modifying the CM induction method from human iPSCs described above. According to our vascular cell differentiation system from mouse ESCs, we induced vascular cells (ECs/MCs) by treating purified Flk-1 positive mesoderm cells with VEGF [36]. We showed that the proportion of mesoderm cells, which possess the potential to differentiate into cardiovascular cell populations, peaks at differentiation day 5 in our CM differentiation system from hiPSCs [53], and we added VEGF from day 5 and to induce CMs and vascular cells simultaneously (CM 67–85 %, EC 8–13 % and MC 3–19 % of total cells; 201B6 line) [38•].

Using the same manner as mouse ESCs and the temperature-responsive culture surface, we successfully collected self-pulsating CTSs from human iPSC-derived cardiovascular populations induced by the differentiation method mentioned above, which mimics endogenous human cardiac tissue. We also tried to generate cell sheets from purified human iPSC-derived CMs, but failed to form the cell sheet structure, indicating the significance of vascular cells. We transplanted a three-layered CTS to a rat MI model and confirmed functional recovery, which was sustained as long as 2 months after transplantation. A histological evaluation revealed detectable engraftment of the transplanted human cells on 44 % of all transplanted rats, with an average of 24.7 % coverage of the MI area 4 weeks after transplantation [38•]. These results indicate the excellent potential of human iPSC-derived CTSs toward cardiac regeneration.

## Human iPSC-derived cardiac tissue bioengineered with CTSs and gelatin hydrogel microspheres

So far, we have introduced our strategy for cardiac regeneration using CTSs. These transplanted CTSs mimic the cardiac tissue structure and exhibit excellent potential for cardiac functional recovery and myocardial regeneration. To achieve further engraftment efficiency as a de novo myocardium in an injured heart and sustained functional recovery, we have also generated much thicker human iPSC-derived cardiac tissue in vitro.

The simplest method to generate thick tissues based on the CTS method would be to stack CTSs in vitro. However, it is reported that a 3-sheet stack (less than 100 μm) is usually the upper limit of stacking due to hypoxic cell damage and shortage of nutrient supply leading to central necrosis [54, 55]. To overcome this stacking limit, we employed gelatin hydrogel microspheres (GHMs), a novel biomaterial reported to support oxygen and nutrient supply, and found they improved cell survival [56]. We applied GHMs between every stacked layer of CTSs to prepare a 15-layer structure of mouse ESC-derived CTSs with >1 mm thickness and enough viability to be cultured in vitro for over 1 week. Transplantation of a 5-layered CTS construct with GHMs to a rat MI model showed potent and sustained functional recovery. The transplanted grafts were engrafted as a multi-layered cardiovascular cell structure and resulted in functional capillary networks and a de novo myocardium (0.8 mm thickness) 3 months after transplantation [57•]. These thick viable cardiac tissues from PSCs are a remarkable technological innovation, as they exhibited sustained functional benefits and survival of the cell graft in injured heart.

Despite these gains, CTSs with GHMs still have several hurdles to overcome before their clinical application. First, we need to verify contractile synchrony between the transplanted cardiac tissue and the underlying myocardium and also any arrhythmogenic potential. Although we have demonstrated electric coupling between the graft and host rat heart by simultaneously recording the extracellular field potential of the cardiac tissue and rat electrocardiogram [57•], these observations should be validated using animal models that have similar intrinsic beating rates to human hearts. Second, it is desirable to improve the vascular connectivity and perfusion of the cardiac tissue in the immediate post transplantation setting considering that the lack of immediate vascular connections and oxygen supply significantly limit the therapeutic efficacy. Bioengineering strategies that pre-vascularize the graft tissue might enhance the vascular connection between the host and graft for better long-term effect [58].

## Conclusion

In this review, we have introduced our strategies for the engraftment of transplanted cardiovascular cells as a de novo myocardium based on PSC-engineered cardiac tissue generation. We anticipate that these innovations will enhance the efficacy of cardiac stem cell therapy and contribute to future cardiac regenerative medicine.
